# Soil property controls on plasticiser, antioxidant and UV absorber additive degradation across a global soil gradient

**DOI:** 10.1007/s11356-025-37152-2

**Published:** 2025-12-04

**Authors:** Michaela K. Reay, Martine Graf, Maddy Murphy, Charlie Monkley, Perrine J. Florent, Benjamin I. Collins, Nguyen Van Hien, Tran Minh Tien, Andreia Neves Fernandes, Tapan Adhikari, Samantha Viljoen, Mona Tolba, Ahmed Mosa, David R. Chadwick, Davey L. Jones, Richard P. Evershed, Charlotte E. M. Lloyd

**Affiliations:** 1 Organic Geochemistry Unit, School of Chemistry, University of Bristol, Bristol, BS8 1TS UK; 2 School of Environmental and Natural Sciences, Bangor University, Bangor, LL57 2UW UK; 3 Soils and Fertilizer Institute, Ha Noi, Vietnam; 4 Federal University of Rio Grande Do Sul, Porto Alegre, Brazil; 5 ICAR-Indian Institute of Soil Science, Bhopal, India; 6 Bioplastics Innovation Hub, Food Futures Institute, Murdoch University, Perth, Australia; 7 Soils Department, Faculty of Agriculture, Mansoura University, Mansoura, 35516 Egypt; 8 Plant Nutrition Department, National Research Centre, Dokki, Giza, Egypt; 9 School of Geography, University of Bristol, Bristol, BS8 1SS UK

**Keywords:** Plasticiser, Antioxidant, UV absorber, Microbial turnover, Abiotic degradation

## Abstract

Additives in agricultural plastics can leach into the surrounding soil during use or improper disposal. Their subsequent degradation rates directly regulate whether they persist and accumulate to levels with ecotoxicological effects or are rendered benign. However, which soil properties primarily regulate the degradation of additives remains unclear (e.g. soil carbon, pH, available nutrients, microbial biomass and community structure). We assessed the degradation of the common plastic additives with different functionalities (DEHP (di(2-ethylhexyl) phthalate; plasticiser), 2-hydroxy-4-n-octyloxybenzophenone (benzophenone-12; BP12; UV stabiliser) and AO168 (tris(2,4-di-*tert*-butylphenyl) phosphite; antioxidant)) in soils under controlled moisture and temperature conditions over 21 days across contrasting agricultural soils from six countries across a global transect (Australia, Brazil, Egypt, India, Vietnam and the UK). DEHP followed zero-order degradation kinetics, with negligible degradation in soils with low microbial biomass. BP12 degraded fastest via first-order degradation kinetics via ether cleavage and hydroxyl loss. The degradation of DEHP and BP12 was correlated with soil microbial biomass and nitrate concentration. BP12 degradation products detected included benzophenone and benzoic acid. DEHP is degraded via β-oxidation of alkyl groups to dibutyl phthalate and diethyl phthalate and through ester hydrolysis to phthalic acid. AO168 degraded via abiotic oxidation and phosphate ester hydrolysis to 2,4-di-*tert*-butyl-phenol, and degradation was not well correlated with any measured soil variable. Overall, these results show that the components of additive mixtures leached into soils will degrade at different rates due to varying mechanisms and controls exerted by the soil microbial biomass. Plastic additives have differing potentials to persist in agricultural soils globally, with some likely to accumulate to levels that may impact soil function and pose an ecotoxicological threat to soil biota.

**Supplementary Information:**

The online version contains supplementary material available at 10.1007/s11356-025-37152-2.

## Introduction

Plastic additives are now ubiquitous in the soil environment (Costa et al. 2023) and are of particular interest in agricultural soils, where there is potential for organic xenobiotics to be taken up into crops and enter the food chain (Gao et al. 2017). Additives present in soils may arise from a range of sources, such as agricultural plastics (e.g. plastic mulch films, irrigation pipe, polytunnels (Briassoulis et al. 2013), agrochemicals (e.g. coated seeds and fertilisers), organic resources (e.g. composts, biosolids) (Diao et al. 2023) or external inputs (e.g. irrigation water, atmospheric deposition (Dueñas-Moreno et al. 2024). Additives with different roles in plastics (Hahladakis et al. 2018) will co-exist in soils due to these different sources. For example, phthalic acid esters (PAEs) (X. Li et al. 2023a, b), antioxidants (Gong et al. 2021) and UV absorbers (Li et al. 2023a, b) are all plastic-derived xenobiotics that have been separately identified in agricultural soils. Some plastic-derived additives identified in soils raise concern with regard to human health and environmental bioaccumulation (Martino‐Andrade and Chahoud 2010; Kim et al.^ 2019^; Wiesinger et al. 2021). For example, PAEs such as di(2-ethylhexyl) phthalate (DEHP), dibutyl phthalate (DBP) and diisobutyl phthalate (DIBP) are harmful to human health as they are known carcinogens and endocrine disruptors (Heudorf et al.^ 2007^). Some antioxidants have also been identified as hazardous for human health and may bioaccumulate in the environment (e.g. tris(2,4-di-*tert*-butylphenyl) phosphite (AO168) and its oxidation product, tris(2,4-di-*tert*-butylphenyl) phosphate (AO168ox)) (Yang et al. 2011; Hammond et al. 2014; Wang et al. 2023). Benzophenone UV absorbers are, in general, toxic to aquatic organisms (Li 2012) and show varying persistence in soils and water, influenced by solubility (Li et al. 2023a, b; Carstensen et al. 2023). In view of these hazards, the potential for additives to accumulate in soils and enter the food chain via flora and fauna uptake (Du et al. 2009; Li et al. 2020) or be transported to aquatic ecosystems is a major area of concern (Hahladakis et al. 2018; Schmidt et al. 2020).

**Fig. 1:**
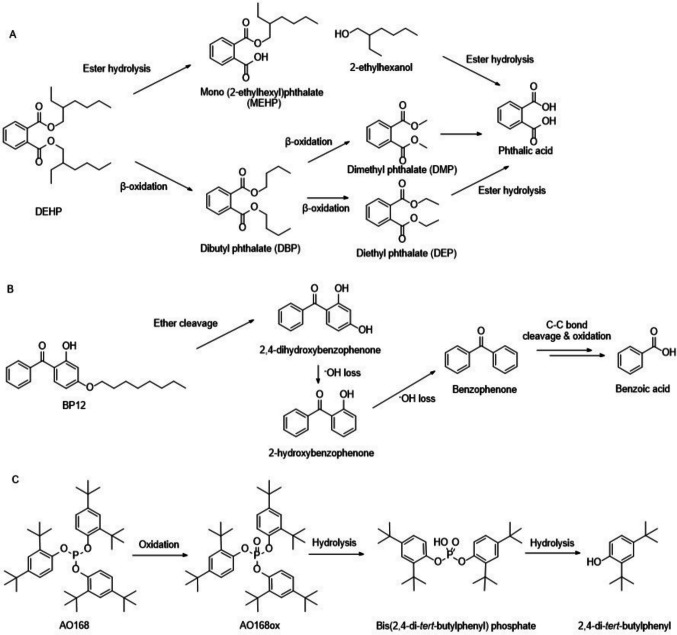
Targeted degradation products of **A** DEHP (Zhu et al. 2018; Tran et al. 2023), **B** BP12 (Badia-Fabregat et al. 2012) and **C** AO168 (Gong et al. 2021)

The impact of plastic additives on soil health is likely to depend on the rate at which they degrade in soil. Additives can be degraded via abiotic mechanisms (e.g. UV irradiation, oxidation); however, the dominant breakdown pathway in most soils is likely to be mediated by the soil microbial community. Microbial degradation of PAEs proceeds via β-oxidation of alkyl chains and enzymatic hydrolysis of the ester bond (e.g. DEHP in Fig. 1 (Zhu et al. 2018), to yield phthalic acid as an intermediate product, which is subsequently degraded via ring cleavage (Hu et al. 2021). At the same time, UV exposure can increase degradation (Viljoen et al. 2023), with only negligible degradation in sterile soil (Xie et al. 2010). Similarly, the degradation of benzophenone UV absorbers proceeds biotically, via ether cleavage, ring-cleavage of benzene rings and hydroxylation (Badia-Fabregat et al. 2012; Jin et al. 2019). While degradation can proceed under sterile conditions, indicating abiotic degradation, this is slower than in nonsterile soils (Li et al. 2023a, b). Organophosphite antioxidant degradation primarily proceeds via oxidation and then hydrolysis (e.g. AO168 in Fig. 1). However, comparable degradation of AO168 and bis(2,4-di-*tert*-butylphenyl) pentaerythritol diphosphate in sterilised and unsterilised soil conditions indicated that this proceeds primarily through an abiotic route (Gong et al. 2021). A crucial limitation of previous work is that they have only quantified the degradation of either single compounds or compounds possessing the same functionality in plastics. This neglects the highly complex mixture of additives typically present in plastic, which are likely to have vastly different degradation rates based on their structures and solubility. Furthermore, previous quantification of degradation rates of additives has largely been conducted in one soil type or for only one or two types of additives of the same type (Zhu et al. 2018). Differences in applied concentrations and degradation conditions pose a challenge for comparing additive degradation rates between studies and determining the effects of soil properties for different types of additives (e.g. C and nitrogen (N) availability, microbial biomass). The potential for degradation rates to vary between soils has been demonstrated for DEHP and DBP, where degradation was slower in an agricultural soil with a higher soil organic matter content (Xu et al. 2008). Furthermore, degradation in 12 agricultural soils demonstrated the role of soil pH on DEHP degradation, which will also feedback on the microbial community size and structure, and the presence of some degradation products (e.g. mono (2‑ethylhexyl) phthalate, MEHP) (Zhu et al. 2018). However, the effect of different soil types on the degradation of other additives has not been elucidated (Xu et al. 2008).

In this study, we aimed to quantify the degradation rates of three common plastic additives and the production of their breakdown products (Fig. 1) in six agricultural soils collected across a global transect to investigate the effect of soil properties on the degradation shortly after additive leaching into soil. Three additives with contrasting roles in plastics and chemical structures to capture potential different controls were selected: DEHP (plasticiser), 2-hydroxy-4-*n*-octyloxybenzophenone (benzophenone-12; BP12; UV stabiliser) and AO168 (antioxidant; Table 1). We investigated correlations between soil pH, available nutrients (ammonium (NH_4_^+^), nitrate (NO_3_^−^), phosphate (PO_4_^3−^)), total C and N and microbial biomass, determined via phospholipid fatty acid (PLFA) analyses, with additive degradation rates. Targeted detection of degradation products was also undertaken (Fig. 1) to determine the potential for production across different soil types. We hypothesise that (i) a larger soil microbial community and higher soil C will result in faster additive degradation, (ii) degradation product production will be correlated with additive degradation and (iii) relative additive degradation rates will be DEHP > BP12 > AO168 due to their differing chemical functionality and mechanisms of degradation.

**Table 1:** Summary of plastic additives used for soil degradation experiments and minimum and maximum concentrations previously observed in agricultural soils. As AO168 degrades during agriplastic production, we include its oxidation product (AO168ox) to prevent underestimation of this additive.

	DEHP	BP12	AO168 & AO168ox
Full name	di(2-ethylhexyl) phthalate	2-hydroxy-4-n-octyloxy benzophenone	tris(2,4-di-tert-butylphenyl) phosphite (tris(2,4-di-tert-butylphenyl) phosphate)
Structure	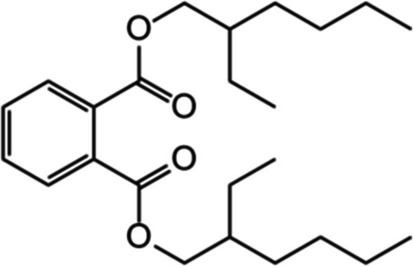	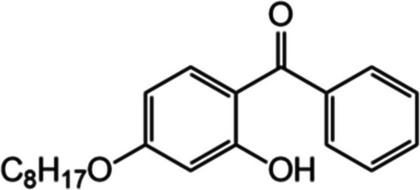	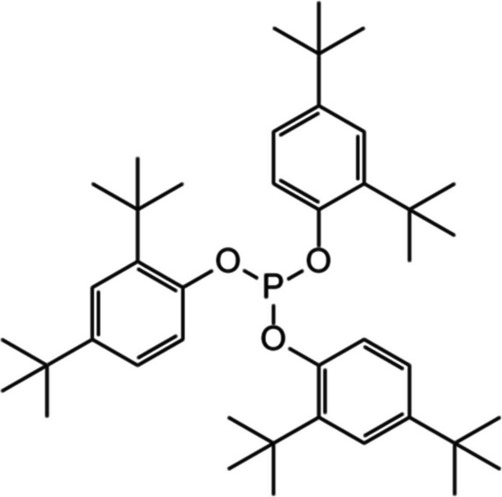
Molecular weight (g mol−1)	390.6	326.4	646.9 (662.9)
Function	Plasticiser	UV absorber	Antioxidant
Log(Kow)	7.76	6.96	11.3
Soil concentration (min-max; μg g−1)	0.03 – 33 (Billings et al., 2021)	0.00012 – 0.00064 (Li et al., 2023)	0 – 0.006 (0 – 0.73) (Gong et al., 2021)

## Materials and methods

### Soil sampling locations

Soil was collected from one location in six countries (Australia, Brazil, Egypt, India, the UK and Vietnam), to capture a sample of representative agricultural soil types across the globe. At each location, 10 cores to a depth of 10 cm were collected and combined as one composite soil sample. The soil was air-dried and then sieved to 5 mm to remove any plant material and homogenise the soil, while minimising potential disruption to fungal networks (Jones and Willett 2006). Soil was then transferred to the UK to undertake the degradation experiment and other soil property analyses. To avoid the influence of past additive inputs, sites were chosen with no known history of plastic use, which was confirmed by analysing additives of interest in all soils, except those in India. Subsequent quantification of DEHP in the Indian soil indicated there was a previously unknown input of this additive at the location; therefore, it was not included in the subsequent data analysis for DEHP or its degradation products. The experimental soil properties are shown in Table 2, and further background information on each location is shown in Table S1. For clarity, each soil is referred to by its country of origin, although we acknowledge that the selected soil will not be fully representative of all soils in each country.

**Table 2:** Initial properties of soils used for additive degradation experiments

Soil property	Australia	Brazil	Egypt	India	UK	Vietnam
Total C (%)	0.7	4.7	0.2	1.1	3.1	1.3
Total N (%)	0.07	0.31	0.04	0.12	0.28	0.12
C:N ratio	10.0	15.1	5.0	9.2	11.1	10.8
Soil respiration (mg CO_2_ kg^−1^)	41	141	6	26	165	133
Sand (%)	83	17	98	17	47	32
Silt (%)	8	30	1	28	25	54
Clay (%)	9	53	1	55	28	14
Soil texture classification	Loamy sand	Clay	Sand	Silty clay	Clay loam	Sandy silt loam
pH	6.09 ± 0.02	7.10 ± 0.01	9.04 ± 0.01	8.21 ± 0.01	6.18 ± 0.04	5.83 ± 0.04
EC (µS cm^−1^)	38.5 ± 3.9	108 ± 7.2	219 ± 23	127 ± 3.2	92.8 ± 3.1	183 ± 5.2
Total PLFAs (mg kg^−1^)	5.30 ± 0.54	30.9 ± 1.58	1.85 ± 0.07	13.5 ± 0.77	28.8 ± 3.2	16.9 ± 1.2
Available P (mg P kg^−1^)	0.20	0.68	0.06	0.12	0.48	0.49

n.b. all concentrations are expressed on a dry weight basis where applicable

### Additive degradation experiments

To ensure homogeneous distribution in soil, additives were added to the soil loaded onto a quartz sand matrix (0.15–0.42 mm, Fisher Scientific). This reflects that hydrophobic additives leached from plastics will be dispersed and initially presented to soil microbes via sorption on organic and/or inorganic particles or colloids/emulsions (Kögel-Knabner and Totsche 1998; Ren et al. 2018). Previously observed concentrations of the selected additives in soil are highly variable and may reflect differences in degradation and inputs (Table 1). The final additive concentration in soil (0.5 µg g^−1^) was selected as it reflects previously observed additive concentrations in soil for DEHP (Billings et al. 2021) and AO168 (Gong et al. 2021), while representing a negligible change in soil carbon, which may feedback on microbial degradation (Table S5). We acknowledge that the concentrations of additives in soils will vary, reflecting differences in inputs and degradation; therefore, this study aims to offer mechanistic insight into controls on degradation and relative degradation of additives, rather than absolute degradation rates, which would also be affected by differences in temperature and soil moisture, alongside other degradation processes (e.g. action of earthworms (Wei et al. 2024), UV (Viljoen et al. 2023). The additives were prepared in dichloromethane (DCM (20 µg mL^−1^ for each additive)), and 0.5 mL was added to the sand carrier matrix (1.0 g, 98% quartz, furnaced at 450 °C to remove any organic C), equivalent to 10 µg g^−1^ of sand. This was then dried at room temperature under a gentle N_2_ flow to remove the solvent, and the sides of the vials were washed with DCM (3 × 0.5 mL) to ensure that additives were transferred to the sand. Extraction tests from the sand (*n* = 10) showed that the recovery of the additives was 98.5 ± 0.5%, 97.9 ± 0.3% and 98.6 ± 0.3% for DEHP, BP12 and AO168, respectively. Sand for the control treatments was prepared in the same way, except additive-free DCM was added.

The quartz sand (1.0 g) was mixed with soil (19.0 g) (giving a 5% *w*/*w* quartz sand content) in glass microcosms with a tapered base plugged with glass wool to ensure aerobic conditions, to give an additive concentration of 0.5 µg g^−1^ soil (Figure S1). The air-dried soil was adjusted to 30% moisture content using HPLC grade water (actual average 28.2 ± 0.6%), determined gravimetrically, which was maintained throughout the experiment. This moisture content was selected to ensure sufficient water availability in each soil type for microbial activity and additive diffusion, while not generating anaerobic conditions. Microcosms were incubated in the dark at 19.4 ± 0.2 °C and 50.3 ± 1.5% relative humidity and destructively sampled at *t* = 0, 1, 2, 3, 7 and 21 days (*n* = 4 for the control and additive treatments). The experimental period used was designed to minimise effects of C limitation in soil-only mesocosms and reflects the dynamics of additives shortly after leaching into soil from plastic when they will be most available to the microbial community. At each sampling timepoint, a sub-sample from the sacrificed microcosm (5 g) was removed for pH and DOC analyses, while the remainder was immediately frozen, then lyophilized for additive and PLFA analyses.

### Soil properties

Initial ammonium (NH_4_^+^) and nitrate (NO_3_^−^) concentrations were determined by extracting soil with 0.5 M K_2_SO_4_ (1:5 *w*/*v*). NH_4_^+^ and NO_3_^−^ concentrations were determined colorimetrically according to the salicylic acid procedure (Mulvaney 1996) and vanadium chloride procedure (Miranda et al. 2001), respectively. Available phosphate was extracted using 0.5 M acetic acid (1:5 *w*/*v*) then quantified using the molybdate blue method (Vaz et al. 1994). Soil texture, total C and N and potential soil respiration were determined by NRM (Bracknell, UK). Soil pH and EC were measured in a 1:2 (*w*/*v*) soil:ultrapure water suspension. Soils were extracted with 0.5 M K_2_SO_4_ (1:5 *w*/*v*), and dissolved organic carbon (DOC) was measured on a Multi-N/C Series TOC/TN analyser (Analytik-Jena, Jena, Germany).

PLFA analyses were conducted on *t* = 0 day and 21 days. Soil (1.0 g) was sonic-extracted using a modified Bligh Dyer solution (15 mL of 2:1:0.8 (*v*/*v*/*v*) methanol, chloroform and KH_2_PO_4_ buffer (0.05 M at pH 7.2)). The total lipid extract in chloroform was then fractionated with activated silica, conditioned with chloroform (5 mL). Neutral lipids were eluted using 5 mL chloroform; glycolipids were eluted using 20 mL acetone, and the phospholipid fraction was eluted with 5 mL methanol, with *n*-nonadecane added as an internal standard. The phospholipid fraction underwent acid-catalysed methylation to obtain fatty acid methyl esters (FAMEs) using 5% (*v*/*v*) HCl in methanol (5 mL, 50 °C for 2 h). The FAMEs were extracted into *n*-hexane (3 × 1 mL), using saturated sodium chloride solution (5 mL) to promote phase separation. PLFAs (in 50 µL hexane) were analysed via GC-FID (flame ionisation detector) and GC-MS for quantification (relative to the internal standard) and identification, respectively. The GC was fitted with a VF23-ms column (60 m, 0.32 µm i.d., 0.15 µm film thickness, Agilent). The temperature programme was 50 °C (1 min) to 100 °C (10 °C min^−1^) to 250 °C (4 °C min^−1^, 15 min hold), with a helium (He) carrier gas flow of 2.0 mL min^−1^. The MS was operated in electron ionisation mode (70 eV) with a full scan range (*m*/*z* 50–650) and a scan time of 0.2 s. Data was acquired and analysed using Xcalibur (version 4.1). Assignment of PLFAs between C_14_ and C_20_ chain length was based on Frostegard et al. (1993) and Joergensen 2022). The sum of firmicutes-derived PLFAs (*i*14:0, *i*15:0, *i*16:0, *i*17:0, *i*18, *a*15:0, *a*16:0, *a*17:0, *a*18:0, *a*19:0) and actinobacteria-derived PLFAs (10Me16:0, 10Me17:0, 10Me18:0) was used to represent Gram-positive bacteria. The sum of cy17:0, cy19:0, 16:1*ω*7, 16:1*ω*9, 17:1*ω*8 and 18:1*ω*7 PLFAs was used to represent Gram-negative bacteria. The sum of 16:1*ω*5c, 18:1*ω*9c, 18:2*ω*6c and 18:3*ω*6,9,12 PLFAs was used to represent fungi, and the 14:0, 15:0, 16:0, 17:0, 18:0, 20:0 and 20:4*ω*6,9,12,15 PLFAs were classed as unspecified.

### Additive extraction and analysis

Additives were extracted from soil (2.0 g) using DCM-acetone (1:1 *v*/*v*; 10 mL) via sonication for 15 min three times. An internal standard (5α-androstane, 30 µg in DCM) was added. The extracts were isolated by centrifugation (3000 rpm, 15 min), transferred then dried at 40 °C with N_2_ (0.6 L min^−1^). Each extraction batch contained a blank, to ensure there was no additive contamination of the extraction solvents or apparatus. The extract was subsequently analysed via GC for quantification (relative to the internal standard) and GC-MS to check for no co-elution with soil-derived compounds. The GC (Thermo Fisher Scientific™ Trace™ 1300 Gas Chromatograph) was fitted with a HP−1 column (100% dimethylpolysiloxane 50 m × 0.32 mm × 0.17 µm, Agilent) and an FID, held at 320 °C. The GC was operated with a constant flow of He (2.0 mL min^−1^) with a PTV inlet (300 °C, split ratio 3:1, splitless time 5 min). The GC oven temperature programme follows: 50 °C (1 min) to 300 °C (15 min) at 5 °C min^−1^. Data acquisition and analysis were performed in Chromeleon® 7 (Version 7.2.1.5833; Thermo Scientific™). The GC-MS (Thermo Fisher Scientific™ ISQ™ LT) was operated under the same conditions as the GC. The MS operated under electron ionisation (70 eV) under a full scan range (*m*/*z* 40–700) at a scan time of 0.2 s. The transfer line to the MS was maintained at 300 °C, and the ion source temperature was set at 300 °C. Data acquisition and analysis used Xcalibur Version 4.1.31.9 (Thermo Fisher Scientific™ Ltd). With each analytical batch, a separate external standard containing the three additives and the internal standard was analysed to determine the relative response of individual additives compared to 5α-androstane for quantification.

Analysis via GC-MS did not detect the presence of degradation products due to the detection limit (LOD) of this technique. Therefore, the extracts were subsequently analysed using an Agilent GC-QTOF-MS. The GC (Thermo Fisher Scientific™ Trace™ 1300 Gas Chromatograph) was fitted with a HP−1 column (100% dimethylpolysiloxane 50 m × 0.32 mm × 0.17 µm, Agilent). The GC was operated with a constant flow of He (1.5 mL min^−1^). The oven temperature programme follows: 50 °C (1 min) to 300 °C (15 min) at 5 °C min^−1^. The MS operated under electron ionisation (70 eV) under a full scan range (*m*/*z* 50–700) at a scan time of 0.2 s. The transfer line to the MS was maintained at 300 °C, and the ion source temperature was set at 300 °C. Data acquisition and analysis used Agilent Masshunter (version B.07.00). Suspected degradation products were also analysed in the same manner to confirm identification and LODs. Quantification was based on an external calibration (15.0 to 1.0 ng µL^−1^), followed by correction for any degradation product observed in the control soil. Limits of quantification are shown in Table S2.

### Contamination control measures

All soil processing and storage were conducted using furnaced aluminium foil (foil, hereafter) to avoid contact with plastic. All microcosms were constructed of glass and covered with foil to prevent any potential dry deposition. All solvents (DCM, methanol, acetone, hexane, chloroform, ethyl acetate, water) were HPLC grade (Rathburn, UK). All compounds were purchased from Merck Life Sciences UK Ltd at 99% purity. All glassware was washed with Decon, double distilled water and acetone, before furnacing at 450 °C for 2 h. Equipment which could not be furnaced was additionally cleaned with DCM, methanol and DCM-methanol (2:1 *v*/*v*). No plastic equipment was used for additive extractions and analyses, except PTFE caps for GC vials, which were confirmed not to transfer any additive contamination.

### Calculations and statistical analyses

All data analysis was performed in R (v4.4.0) (R Core Team 2024). Normality of the data was determined by the Shapiro-Wilk test (*p* ≤ 0.05), and homogeneity of variance of the data was visually checked using residuals *vs*. fitted plots. One-way ANOVAs were used to test differences between soil variables (*p* ≤ 0.05). Principle component analysis (PCA) was used to determine the relative importance of soil variables in differentiating soils used in this study. DOC, pH and EC were quantified across the experimental period, and the effect of treatment (control or additive) and time was determined using mixed effects models, using the mixed function in the afex package (Singmann et al. 2024), with treatment loading as a fixed effect and time as a random effect, and *p* values calculated using the Kenward-Roger method. Models were checked for normality, via QQ plots, and heteroscedasticity was checked by plotting residuals *vs*. fitted values.

Zero-order (Eq. 1, where *k*_*0*_ is the zero-order rate constant (µg g^−1^ day^−1^), *t* is time (day) and [*C*]_0_ is the initial additive concentration (µg g^−1^)) and first-order (Eq. 2); where [*C*]_0_ is the initial additive concentration (µg g^−1^), *k*_*1*_ is the first-order rate constant (day^−1^) and *t* is time (day)) degradation models were fitted to obtain degradation rate constants.

1 $${\left[C\right]}_{t}={{k}_{0}t+[C]}_{0}$$

2 $${\left[C\right]}_{t}={[C]}_{0}{e}^{-{k}_{1}t}$$

Given the timescale of the experiment, the degradation rates presented represent initial degradation kinetics. Zero-order regressions were fitted using the lm function to obtain *k*_*0*_, and first-order regressions were fitted using the nlsLM function (minpak.lm) to obtain *k*_*1*_ (Elzhov et al. 2023). Prior to fitting first-order regressions, ln[additive] against time was used to confirm linearity. The models were compared using the Akaike information criterion (AIC), visual inspection of residuals and normality of residuals (via QQ plots) to select the model with the best fit. As resultant rate constants have differing units (*k*_*0*_ = µg g^−1^ day^−1^ and *k*_*1*_ = day^−1^), for individual additives, if one soil followed different degradation kinetics, the rate constant was converted to a pseudo-rate constant to permit comparison. For DEHP, the UK soil followed first-order kinetics but was converted to pseudo zero-order (*k*_*1*_ × [*C*]_0_). For BP12, the Egyptian soil followed zero-order kinetics and was considered pseudo first order for comparisons between soils (*k*_*0*_/[*C*]_0_). To compare between additives, degradation at 21 days is used. Correlations between soil variables and additive degradation were determined using Pearson correlation coefficients, and correlation networks for additive rate constants were subsequently built using the Pearson correlation index with a significance threshold defined at *p* ≤ 0.05.

## Results

### Initial soil nutrient availability and microbial biomass

The soil pH ranged from 5.8 to 9.0 across the soils and was not affected by the introduction of additives compared to the control treatment (Figure S2). Soil pH and EC (Figure S3) did change (range −0.06 (Brazil) to 0.62 (Vietnam) for pH; −16.7 to 122 µS cm^−1^ for EC) over the experimental period for some soils in the opposite direction (Table S3), although there was no difference between the control and additive treatments (Table S3). Changes in pH and EC likely reflect changes in microbial processes (e.g. nitrification) due to disturbance from sand addition and rewetting, rather than the effect of additive addition. Soil DOC significantly varied between soils (*p* < 0.001; Table 3) and was positively correlated with total soil C (*p* = 0.007), as well as microbial biomass, determined via PLFAs (*p* = 0.038). The total microbial PLFA biomass significantly varied between all soils (*p* < 0.001), as did all contributing groups (Table 3 and Table S4), with soil from Brazil and the UK having the highest microbial biomass. In contrast, the Egyptian and Australian soil had the lowest microbial biomass. Alongside differences in the absolute microbial community size, the composition of the microbial community varied between the soils. The ratio of Gram+ to Gram− bacteria was significantly different between soils (*p* = 0.012), with higher Gram+ bacteria abundance relative to Gram− bacteria in the Vietnamese soil compared to the Egyptian, Australian and Indian soil. The ratio of fungi to bacteria also significantly varied (*p* = 0.004), and the Egyptian soil had higher relative fungal biomass to bacterial biomass, as the Gram+ and Gram− communities were small, although the Egyptian soil had the lowest bacterial and fungal biomass of all soils (both *p* < 0.001).

**Table 3:** Soil properties at *t* = 0 day for each soil. Values indicate mean ± SE (*n* = 4). Significant differences between soils determined via one-way ANOVAs are shown by bold *p* values, and differences between soils, determined by post-hoc Tukey tests, are indicated by different superscript lowercase letters

Soil property	*p*	Australia	Brazil	Egypt	India	UK	Vietnam
DOC (mg kg^−1^)	** < 0.001**	198 ± 63^b^	736 ± 157^a^	123 ± 50^b^	240 ± 63^b^	394 ± 95^ab^	192 ± 17^b^
Available NH_4_^+^ (mg kg^−1^)	** < 0.001**	1.78 ± 0.12^c^	14.0 ± 0.82^b^	2.15 ± 0.11^c^	1.05 ± 0.24^c^	2.81 ± 0.16^c^	19.2 ± 0.5^a^
Available NO_3_^−^ (mg kg^−1^)	** < 0.001**	2.68 ± 0.38^c^	7.87 ± 1.25^b^	1.41 ± 0.09^c^	3.55 ± 0.13^bc^	19.3 ± 2.0^a^	5.09 ± 1.06^bc^
pH	** < 0.001**	6.09 ± 0.02^d^	7.10 ± 0.01^c^	9.04 ± 0.01^a^	8.21 ± 0.01^b^	6.18 ± 0.04^d^	5.83 ± 0.04^e^
Gram + bacteria PLFAs (mg kg^−1^)	** < 0.001**	1.47 ± 0.17^d^	13.1 ± 0.7^a^	0.088 ± 0.008^d^	4.44 ± 0.46^c^	11.0 ± 0.72^a^	6.98 ± 0.52^b^
Gram- bacterial PLFAs (mg kg^−1^)	** < 0.001**	1.10 ± 0.15^c^	8.02 ± 0.49^a^	0.077 ± 0.008^d^	3.33 ± 0.14^bc^	7.07 ± 1.96^ab^	2.97 ± 0.23^c^
Fungal PLFAs (mg kg^−1^)	** < 0.001**	0.88 ± 0.14^c^	5.75 ± 0.34^a^	0.18 ± 0.04^c^	2.57 ± 0.2^4b^	6.65 ± 0.51^a^	2.70 ± 0.22^b^
Bacterial PLFAs (mg kg^−1^)	** < 0.001**	2.57 ± 0.31^ cd^	21.2 ± 1.12^a^	0.17 ± 0.01^d^	7.77 ± 0.41^bc^	18.1 ± 2.6^a^	9.95 ± 0.75^b^
Total PLFAs (mg kg^−1^)	** < 0.001**	5.30 ± 0.54^c^	30.9 ± 1.58^a^	1.85 ± 0.07^c^	13.5 ± 0.77^b^	28.8 ± 3.2^a^	16.9 ± 1.2^b^
Gram+:Gram− ratio	**0.012**	1.36 ± 0.04^b^	1.64 ± 0.06^ab^	1.18 ± 0.17^b^	1.35 ± 0.18^b^	1.94 ± 0.47^ab^	2.35 ± 0.03^a^
Fungal:bacterial ratio	**0.004**	0.34 ± 0.03^b^	0.27 ± 0.01^b^	1.13 ± 0.35^a^	0.33 ± 0.02^b^	0.39 ± 0.05^b^	0.27 ± 0.01^b^

n.b. all concentrations are on a dry weight basis and corrected for the mass of added sand

**Fig. 2:**
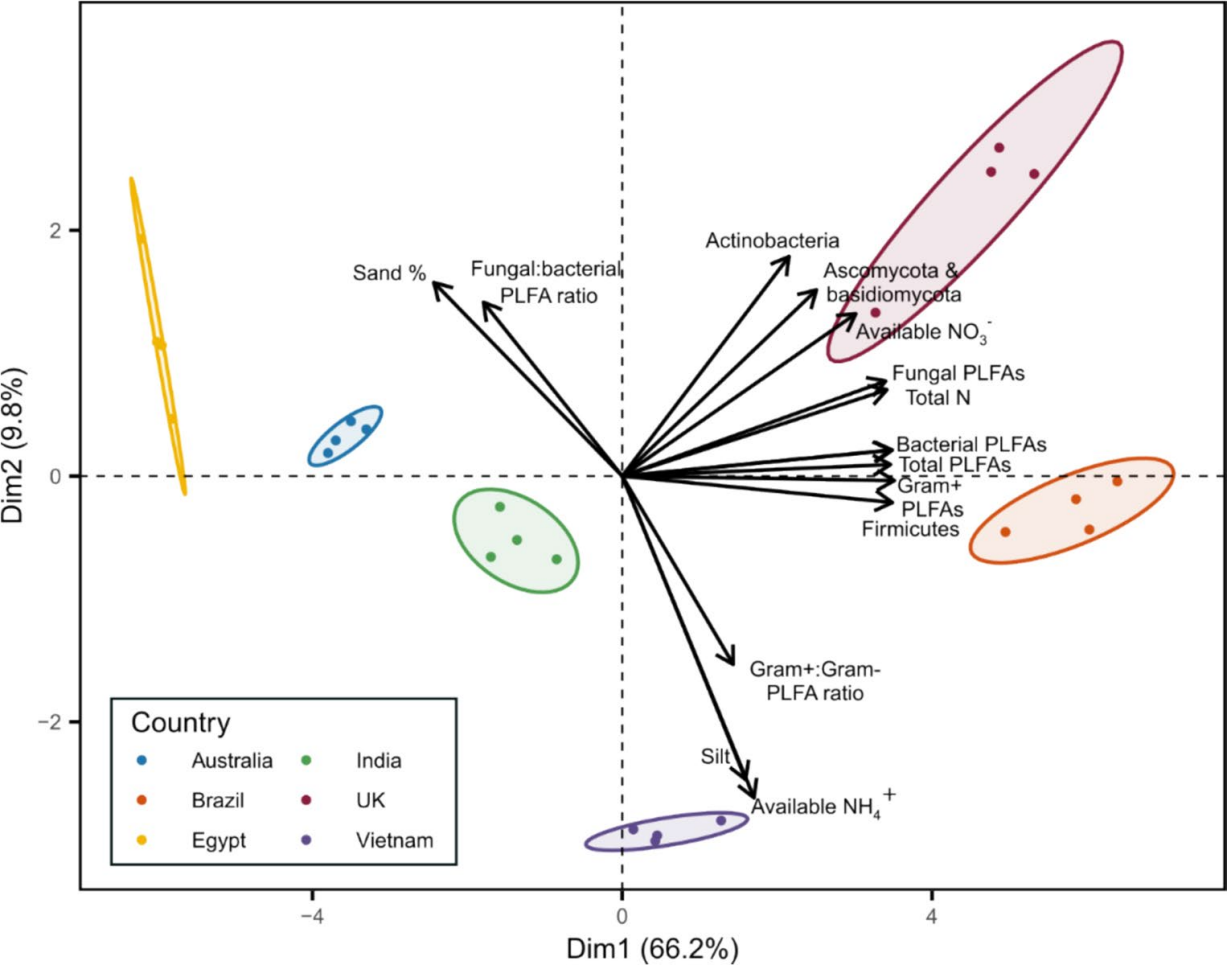
Principal component analysis biplot of initial soil properties for soils used in additive degradation experiments. The arrows indicate the variables that had a significant contribution to the PCA analysis, defined as a loading of > 0.5, with the relative contribution indicated by arrow length. Ellipses represent 95% confidence intervals for each country, and individual points indicate replicates (*n* = 4)

Soil properties (Table 2), initial nutrient availability and microbial biomass parameters (Table 3) were combined to elucidate the parameters driving differences between the soils via PCA analyses (Fig. 2). The PCA was built over the first two dimensions, together covering 76% of the fitted variation. The majority of the differences between soils were in Dim1 (66.2%, Fig. 2) and were driven by the microbial community size. The parameters with significant contributions to Dim1 included the total microbial biomass (5.2%), Gram+ bacteria (5.3%) and contributing bacterial groups (Firmicutes (5.2%)). Total fungal PLFAs (5.0%) and Ascomycota and Basidiomycota (2.7%) contributed significantly to Dim1. Soil nitrogen, namely total N and available NO_3_^−^, was also a significant driver of differences between soils in Dim 1 (5.0% and 3.9%, respectively). Differences between soils in Dim2 (9.8%) were influenced by the microbial community structure, indicated by the contribution of the Gram+:Gram− ratio and fungal:bacterial ratio (6.8% and 5.8%, respectively). In comparison, soil texture (7.2%) and available NH_4_^+^ (9.9%) also contributed to the differences observed in Dim2. Overall, the properties of the soil significantly varied across the global transect, providing a platform to determine the effects of soil properties on additive degradation dynamics.

### Soil DOC dynamics during additive degradation

**Fig. 3:**
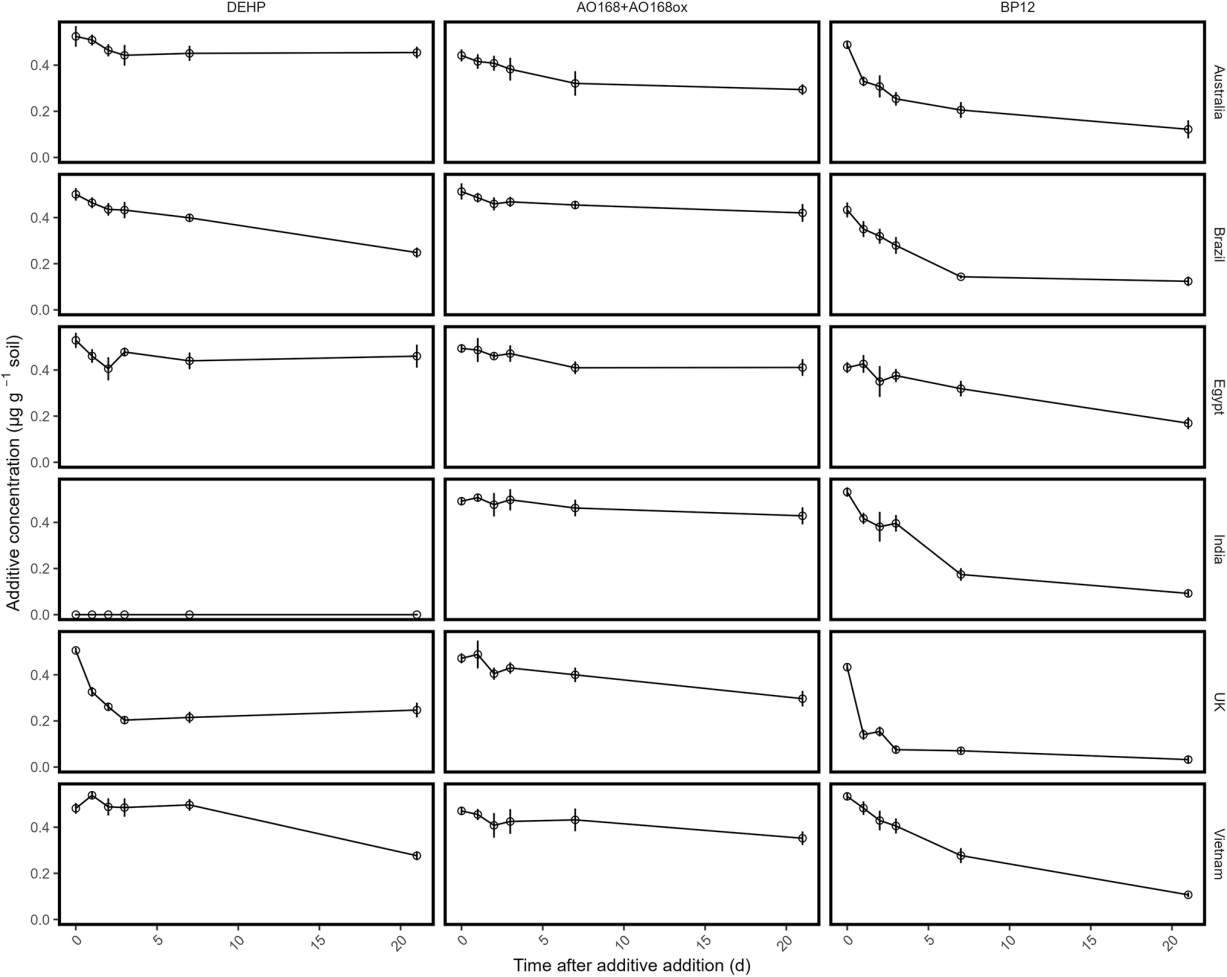
Degradation of the plastic additives DEHP, AO168 + AO168ox and BP12 over 21 days across a global range of soil types. Values are mean ± SE (*n* = 4). Note that DEHP degradation in the Indian soil was not determined due to high background levels of this additive at the .

Changes in soil DOC did not vary between the control and additive treatments (Figure S4, Table S3). However, soil DOC did decrease across the experimental period for all soils, as soil microbes utilised available C, and there were no additional plant C inputs (e.g. root exudates). In some soils (Australia, Egypt, India and Vietnam), there was an initial increase in DOC, which may be an artefact of rewetting and mixing of the soil when the sand was added. With respect to total C, the additive contribution was 0.016 ± 0.008% (range = 0.002 to 0.057%; Table S5), and additives contributed 0.50 ± 0.10% (range = 0.15 to 0.92%) of DOC. Therefore, the additive addition to the soil did not significantly alter C availability at *t* = 0 day. As DOC decreased during the experiment, despite degradation, the additive contribution to soil DOC increased (1.88 ± 0.51% at *t* = 21 days), with higher contributions in soils with lower DOC (e.g. Egypt 4.26%). However, higher contributions to soil DOC were not reflected in the degradation of additives (Fig. 3).

### Additive degradation rates

The degradation of the three additives across the soils followed the order of BP12 > DEHP > AO168 + AO168ox for the Brazilian, the UK and Vietnamese soils and Australian and Egyptian soils BP12 > AO168 + AO168ox > DEHP, based on degradation at 21 days. For the Indian soil, where DEHP degradation could not be determined, degradation at 21 days was BP12 > AO168 + AO168ox (Fig. 3). The degradation of BP12 followed first-order kinetics in all soils except Egypt, which displayed zero-order kinetics, and all exhibited significant degradation across the time course (all *p* < 0.001; Table 4). The degradation rate was fastest in the UK soil (*k*_*1*_ = 0.671 day^−1^), indicating this additive does not persist in this soil. In contrast, the other soils had slower degradation (*k*_*1*_ = 0.080–0.122 day^−1^), including for the Egyptian soil.

**Table 4:** Additive degradation following additive addition to soil over 21 days. The kinetic rate constant (*k*_*0*_ or *k*_*1*_) for each additive and soil combination is the best model fit determined via comparison of AIC and residuals. Rate constants in brackets represent pseudo rate constants used to determine additive degradation rates correlated with soil properties (pseudo-*k*_*1*_ = *k*_*0*_/[*C*]_0_ and pseudo-*k*_*0*_ = *k*_*1*_ × [*C*]_0_). *p* values are shown for the best model fit, and significant *p* values are indicated in bold (*p* ≤ 0.05). India was excluded from DEHP analyses due to high concentrations of DEHP in the collected soil

Country	DEHP				AO168 + AO168ox			BP12			
	Degradation at 21 d (%)	***k***_***0***_ (µg g^−1^ d^−1^)	***k***_***1***_ (d^−1^)	***p***	Degradation at 21 d (%)	***k***_***0***_ (µg g^−1^ d^−1^)	***p***	Degradation at 21 d (%)	***k***_***0***_ (µg g^−1^ d^−1^)	***k***_***1***_ (d^−1^)	***p***
**Australia**	9.0 ± 1.7	0.0021	-	0.24	41.2 ± 3.4	0.0065	**0.002**	75.6 ± 5.2	-	0.096	** < 0.001**
**Brazil**	50.3 ± 3.1	0.011	-	** < 0.001**	21.5 ± 2.3	0.0034	**0.018**	75.3 ± 2.9	-	0.110	** < 0.001**
**Egypt**	13.3 ± 2.6	0.0009	-	0.68	24.0 ± 2.2	0.0037	**0.033**	66.1 ± 3.9	0.011	(0.022)	** < 0.001**
**India**	-	-	-	-	20.0 ± 1.6	0.0034	0.063	81.6 ± 1.6	-	0.122	** < 0.001**
**UK**	50.6 ± 2.9	(0.015)	0.030	**0.043**	40.7 ± 2.4	0.0060	** < 0.001**	93.5 ± 2.3	-	0.671	** < 0.001**
**Vietnam**	44.7 ± 1.7	0.011	-	** < 0.001**	29.6 ± 2.5	0.0046	**0.028**	78.6 ± 1.1	-	0.080	** < 0.001**

Degradation of DEHP followed zero-order kinetics, with linear degradation across the experimental period (Table 4, Fig. 3), except for the UK soil, which exhibited first-order degradation kinetics, with rapid degradation of DEHP to *t* = 3 days, and a subsequent plateau to 21 days. While the UK soil showed different degradation kinetics, the degradation at 21 days was comparable at 21 days for the UK, Brazilian and Vietnam soils (*p* > 0.05). The Egyptian soil had the slowest degradation (*k*_*0*_ = 0.0009 µg g^−1^ day^−1^), while the Australian soil also had slower degradation compared to other soils (*k*_*0*_ = 0.0021 µg g^−1^ day^−1^), and the fitted regression models were not significant (see Table 4), indicating there was minimal degradation across the 21-day experiment.

AO168 is presented as the sum of AO168 and its oxidised form, AO168ox, as oxidation to AO168ox was observed during extraction in initial tests. AO168 is also known to oxidise to AO168ox during plastic manufacturing (Haider and Karlsson 2002). Therefore, a mixture of the two is also expected in plastic entering soil. In all soils, the degradation of AO168 + AO168ox operated under zero-order kinetics, and there was significant degradation in all soils except India (*p* = 0.063; Table 4). Australia had lower degradation rates for the other additives, particularly for DEHP. However, the degradation rate of AO168 + AO168ox was highest in this soil (*k*_*0*_ = 0.0065 µg g^−1^ day^−1^, *p* = 0.002), and comparable to the UK (*k*_*0*_ = 0.0060 µg g^−1^ day^−1^). Similarly, while the Brazilian soil showed high degradation rates for BP12 and DEHP, the degradation rate (*k*_*0*_ = 0.0034 µg g^−1^ day^−1^) was comparable to the Egyptian and Indian soil.

### Additive degradation products

**Fig. 4:**
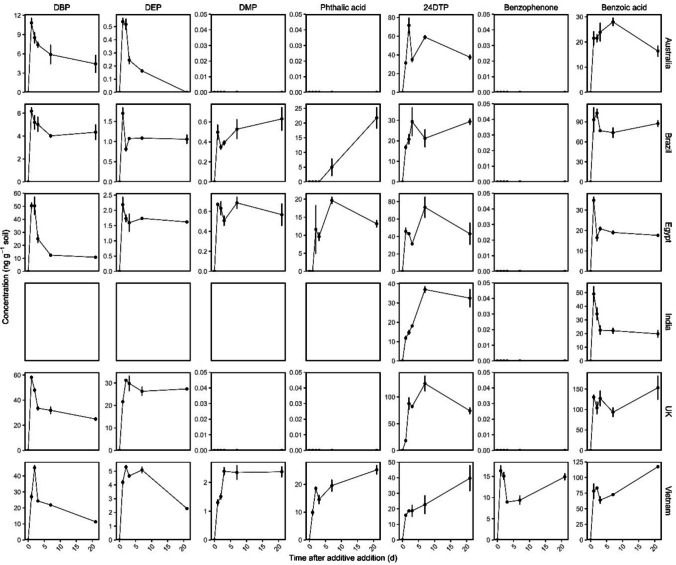
Degradation products of DEHP (DBP, DEP, DMP and phthalic acid), AO168 + AO168ox (24DTP) and BP12 (benzophenone, benzoic acid) after additive addition. Values are mean ± SE (*n* = 4). Note that DEHP degradation products in the Indian soil are not presented due to high background levels of this additive at the site, indicated by no y-axis shown. Possible degradation products not detected (outlined in Fig. 1) are also not presented

Targeted degradation products of additives detected in soils are shown in Fig. 4. For DEHP, the breakdown products DBP, DEP, DMP and phthalic acid were detected in soils from different countries; however, other degradation products (MEHP) were not detected. The Indian soil was excluded from this comparison due to other potential sources of PAEs detected in the control soils. DBP concentration peaked 1 day after additive addition, then declined, and was the most abundant degradation product observed across all soils. A similar trend was observed for DEP and DMP (where observed). In the soils where phthalic acid was detected (Brazil, Egypt and Vietnam), the highest concentration of DBP and DEP was observed later (7 or 21 days). The sum of all DEHP degradation products was negatively correlated with the remaining DEHP in soil (*p* = 0.003) at 21 days (Figure S5a).

The only degradation product of AO168 and AO168ox detected was 2,4-di-*tert*-butyl-phenol (24DTP), which increased in abundance in all soils after addition. A later decline observed in Australia, Egypt and the UK soils suggests production < degradation of 24DTP, while production was comparable to degradation for the Brazilian and Indian soils. Production of 24DTP from AO168 was continually higher than further degradation in the Vietnam soil, indicated by the continued increase in 24DTP concentration until 21 days. 24DTP was negatively correlated with the remaining AO168 concentration at 21 days (*p* = 0.035; Figure S5b).

In the case of BP12, the breakdown product benzophenone was only detected in one soil, Vietnam, where it rapidly increased within 1 day of additive addition and then plateaued to 21 days. Benzoic acid was detected in all soils, with maximum production observed between 1 and 7 days. Subsequently, differing trends were observed of either comparable production and degradation (Brazil, UK), greater production of benzoic acid compared to further degradation (Australia, Egypt, India) or, in the case of the Vietnamese soil, continued production of benzoic acid until 21 days after additive addition. The sum of benzoic acid and benzophenone was negatively correlated with the remaining BP12 in soil (*r*^*2*^ = 0.31; *p* = 0.017; Figure S5c). No other degradation products of BP12 (Fig. 1) were detected in any soil.

### Effects of soil properties on additive degradation

**Fig. 5:**
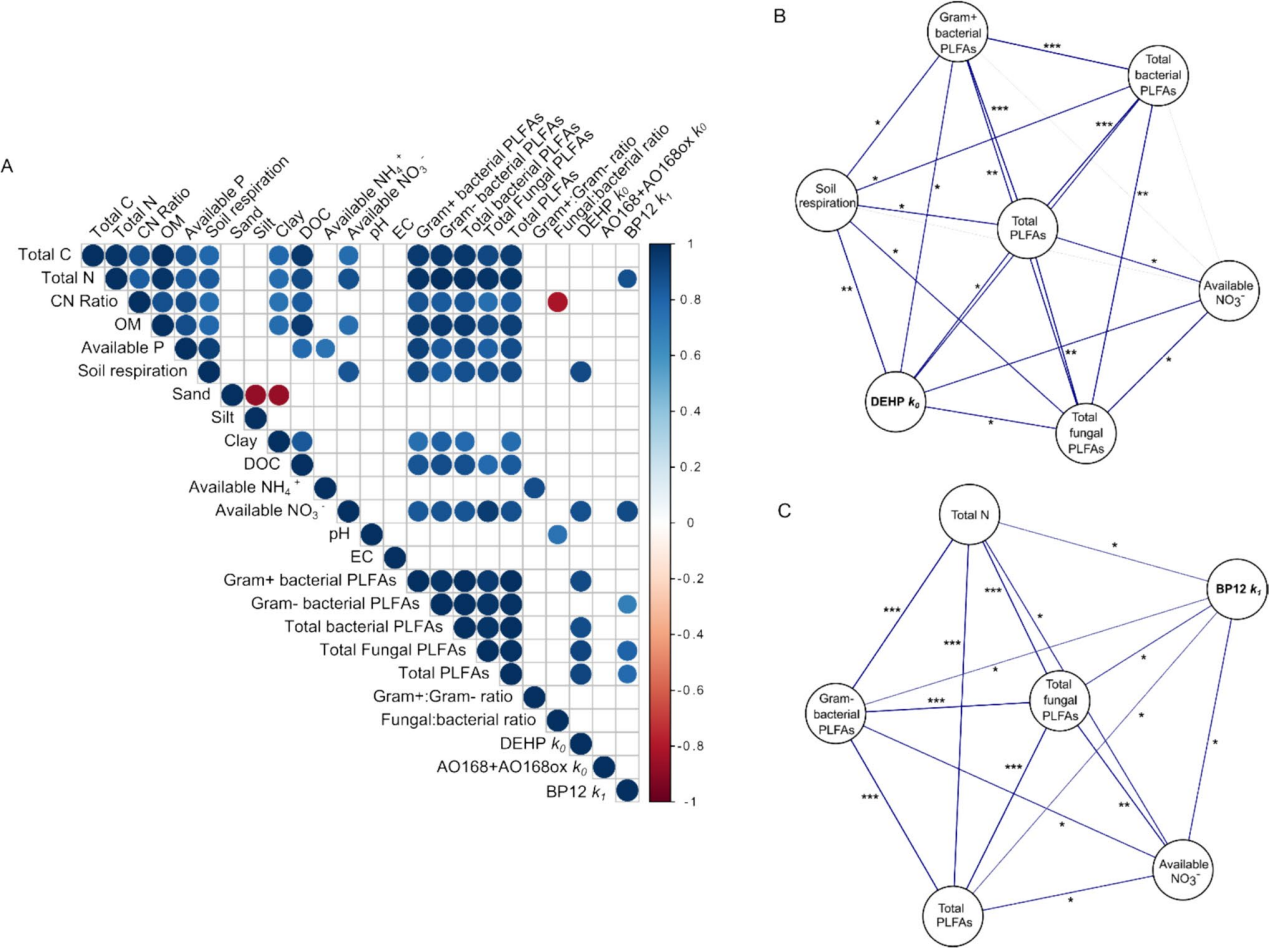
Correlations of soil properties and additive degradation rates (*k*). **A** Pearson correlation matrix, **B** correlation network for DEHP with soil properties, and **C** correlation network for BP12 with soil properties. No correlation network is shown for AO168 + AO168ox as it was not significantly correlated with any soil properties. Significant (*p* ≤ 0.05) positive and negative correlations are indicated by blue and red circles (**A**) or lines (**B**, **C**), respectively. Grey lines in **B** and **C** indicate the correlation between soil parameters was not significant. In **B** and **C**, *p ≤ 0.05, **p ≤ 0.01 and ***p ≤ 0.001

For all additives, the rate constants generally followed the order of UK < Brazil < Vietnam = India < Australia < Egypt (Table 4), and correlations of additive rate constants and soil properties were used to determine the controls of soil type on degradation, alongside the correlation between soil variables (Fig. 5A).

DEHP *k*_*0*_ was positively correlated with total microbial biomass (*p* = 0.019), Gram+ bacterial biomass (*p* = 0.016) and both total bacterial (*p* = 0.019) and fungal (*p* = 0.010) biomass. The positive correlation of the DEHP degradation rate with soil respiration potential (*p* = 0.016) (Fig. 5B) indicated that with a larger and more active microbial biomass, the degradation of this additive increased. DEHP *k*_*0*_ was also positively correlated with available nitrate (*p* = 0.022), but there was no correlation with available C (indicated via DOC, *p* = 0.33). Similar to DEHP, BP12 (Fig. 5C) degradation was also positively correlated with total microbial biomass (*p* = 0.033), and its taxonomic components (Gram− *p* = 0.029 and fungal *p* = 0.045), although it was not correlated with potential soil respiration (*p* > 0.05). BP12 degradation was also positively correlated with available NO_3_^−^ (*p* = 0.017) and total N (*p* = 0.43) but was not correlated with available NH_4_^+^, or determined soil C and P properties. There was no correlation for the *k*_*0*_ rate constant for AO168 + AO168ox with any soil properties (Fig. 5A); therefore, a correlation network was not constructed for this additive.

## Discussion

This study quantified the degradation of three different common plastic additives across a global transect of soils to determine which properties most influence additive degradation and the production of degradation products. Previous studies have either focused on one soil type and/or one additive or additive class; therefore, the findings presented herein represent new insights into the potential chemical burden posed by additives in a range of agricultural soils. Furthermore, by applying a mixture of additives with distinct chemical functionalities, introduced to confer specific properties in plastics, any effects of the co-occurrence of additives on the degradation rates of individual additives are encompassed.

### Biotic controls on BP12 and DEHP degradation

Degradation rates of BP12 and DEHP were both linked to microbial biomass, reflecting the biotic role in ether cleavage and ester hydrolysis for these additives. In this study, BP12 consistently exhibited the fastest degradation rate compared to DEHP and AO168, partially contradicting our original hypothesis regarding the relative degradation rates of the three additives (DEHP > BP12 > AO168). Additives were loaded onto sand to facilitate homogenous distribution of the hydrophobic additives throughout the soil and minimise changes in soil moisture from the addition. The addition of BP12 on a silica matrix in a modified OECD biodegradability test (301F; (OECD 1992)) revealed up to 80% degradation in activated sludge compared to 0% with no silica matrix (Takekoshi et al. 2021) alongside higher degradation in soil than water in other studies (Carstensen et al. 2023; Li et al. 2023a, b). Introducing additives into soil on a sand matrix likely increased degradation rates, particularly for BP12, by promoting microbial accessibility, potentially reflecting the different mechanism of degradation with DEHP (ether cleavage *vs.* ester hydrolysis; Fig. 1). This mode of addition also mirrors how hydrophobic additives leached from plastics will be dispersed and presented to soil microbes via sorption on organic and/or inorganic particles or colloids/emulsions (Kögel-Knabner and Totsche 1998; Ren et al. 2018). Similar to BP12, DEHP possesses low water solubility (log *K*_OW_ = 7.76; Table 1 (Net et al. 2015)) and is rapidly sorbed to soil particles (Jin et al. 2015). Therefore, microbial degradation of DEHP and BP12 likely occurred close to or on the surface of the sand particles, and larger microbial biomass was correlated with higher degradation of both BP12 and DEHP. Microbes facilitate the biodegradation of sorbed compounds by excreting extracellular polymeric substances that contain extracellular degrading enzymes (Johnsen and Karlson 2004). Formation of a microbial biofilm within 5–36 h in an *Arthrobacter* sp. culture on vermiculite showed high potential to rapidly degrade DEHP (Wen et al. 2016), supporting the suggestion that BP12 and DEHP were degraded following rapid formation of a biofilm on the sand.

The degradation kinetics of BP12 adsorbed to sand were first order in all soils, except the Egyptian soil, where degradation was slowest. The observed degradation kinetics were similar to those of BP12 degradation in a Chinese agricultural soil (Li et al. 2023a, b) and other benzophenones in soil, such as 2-hydroxy-4-methoxybenzophenone (Gautam et al. 2022). When added to a solid inert matrix, the degree of degradation for BP12 was comparable to more water-soluble benzophenones (e.g. 2,4-dihydroxybenzophenone, 2,2′-dihydroxy-4-methoxybenzophenone, 2-hydroxy-4-methoxybenzophenone (Carstensen et al. 2023)) in river water, demonstrating the capacity of microbes to degrade benzophenones in the environment. Degradation products of BP12 was inferred from oxybenzone (BP3) and was observed to proceed to benzophenone and benzoic acid, but intermediates produced from ether cleavage (2,4-dihydroxybenzophenone) and subsequent OH loss (2-hydroxybenzophenone) were not observed (Fig. 1). These were either not major degradation products or were rapidly further degraded, thus were below detection limits, even in soils where degradation of BP12 was slow. Benzophenone has minimal associated risk and can further degrade relatively rapidly in environmental matrices (Carstensen et al. 2023). The degradation products of BP12 accounted for 3.3 to 30.6% of that added, while 66.1 to 93.5% of BP12 was degraded, indicating further degradation beyond the targeted compounds, and likely assimilation into microbial biomass. While degradation was slower in soils with lower microbial biomass, degradation was relatively high over the 21-day period in all soils, indicating minimal risk of BP12 accumulation in agricultural soils at this concentration.

DEHP degradation rates were also positively correlated with microbial biomass and activity, inferred from potential respiration. Previous studies in agricultural soils from China focussed only on PAEs and also indicated correlation of degradation of DEHP with microbial biomass (Xu et al. 2008), particularly the bacterial contribution (Zhu et al. 2018). The sum of DEHP degradation products detected (DBP, DEP, DMP and phthalic acid) correlated with degraded DEHP cumulatively by 21 days, and increases in DBP and DEP were observed for all soils. Therefore, microbial β-oxidation of the alkyl chains dominated DEHP degradation in all soils (Liang et al. 2008; Li et al. 2019), with hydrolysis of esters, either from DEHP or degradation products, producing phthalic acid (Fig. 1). The intermediate of DEHP degradation to phthalic acid directly via ester hydrolysis, MEHP (Chen et al. 2023), was not observed. Previously, this has only been detected in low pH soils (pH 5.03–5.33 (Zhu et al. 2018), whereas all soils herein had pH values > 5.83, reflecting the agricultural context. Here, MEHP was not a major degradation product or was a rapidly degraded intermediate at the soil pH range studied. While final concentrations of degradation products after 21 days correlated with degraded DEHP, initial production was more variable, potentially due to different rates of consumption of degradation products vs. production, as DBP and DEP can degrade faster in soils than DEHP (Cheng et al. 2018). The decline in DBP and DEP concentration later in the experimental period supports this. DBP and DEP are both endocrine disruptors, as is DEHP, and their production from DEHP therefore may result in negative effects on the soil microbial community (Cheng et al. 2018) and potentially further up the food chain after plant uptake (Gao et al. 2017). Further work to determine the fate of degradation products is needed to determine their potential to persist and the controls of soil properties, particularly at different concentrations to reflect different inputs, either externally or arising from different degradation rates.

There was a large disparity between detected degradation products (0.9–10.5% of added DEHP) compared to DEHP degradation (9.0–50.6%), which supports further degradation of the initial products by microbial utilisation in all soils. This is consistent with the correlation of DEHP with both microbial biomass, including both bacterial and fungal groups within, and microbial activity, inferred from potential soil respiration, and previous observations of negligible degradation under sterilised soil conditions (Xu et al. 2008). In dark conditions, soils with low microbial biomass (e.g. Australian and Egyptian soils) showed potential for accumulation of DEHP as biotic degradation was not significant (Table 4), with a potential influence of higher water filled pore space in coarse textured soils herein (83.8% and 86.4%, respectively). The initial degradation kinetics observed herein followed zero-order degradation kinetics and a dependency on the initial additive concentration, potentially due to saturation of microbial degradation capacity following an initial input of additives, or changes in sorption altering microbial accessibility. The short experimental period was designed to prevent the closed system from becoming nutrient limited and to provide potential feedback on the microbial community. Concerning the UK soil, degradation determined via mineralised ^14^C-labelled DEHP over 4 months in the same soil was only 8.6% (Viljoen et al.^ 2023^), compared to 41% after 21 days here. ^14^C remaining in soil may also include degradation products (e.g. DBP, DEP, phthalic acid) or microbial biomass, having not been completely mineralised to CO_2_. At 21 days, for the UK soil, degradation products accounted for 21% of degraded DEHP (10.5% of total added). Further work using ^13^C-labelled DEHP would reveal the fate of unaccounted for DEHP in microbial biomass.

Aside from the relationship with microbial biomass, both DEHP and BP12 degradation were positively correlated with available NO_3_^−^, but not other nutrients, while BP12 was correlated with total soil N. The addition of fertilisers to soil can enhance PAE degradation in soil by increasing soil microbial biomass (Zhou et al. 2022), and increases in N and P in coastal sediments increased DBP degradation (Zhang and Chi 2020). Findings here show a complex interaction of microbial biomass and nutrient availability influencing initial additive degradation, and further work on how N added as fertiliser to agricultural soils will feedback on additive degradation is required. Changes in additive availability due to sorption aging should also be considered, as this will feedback on longer-term degradation and accumulation potential. The lack of correlation with soil C parameters (total C, OM and DOC) suggested that C limitation of the microbial community did not directly limit degradation, although it is likely that C availability in the soil itself controlled the size of the microbial community, and thus its capacity to degrade additives. DEHP degradation rates have previously been linked to soil C content in a range of agricultural soils (Zhu et al. 2018) and C inputs (Viljoen et al. 2023). This is also the case for soil pH, which did not directly correlate with DEHP, as previously observed (Zhu et al.^ 2018^), or BP12 degradation, but may influence the bacterial diversity (Zhou et al. 2024), hydrolase activity (Zeng et al. 2001) and additive sorption, thus indirectly influencing degradation.

### Abiotic degradation of AO168 was not influenced by soil properties

AO168 is an extensively used organophosphite antioxidant, and in soil, AO168 underwent rapid oxidation to AO168ox, which also occurs during plastic manufacture (Haider and Karlsson 2002). Further degradation of this antioxidant was generally slower than that of the other additives studied herein in soils with higher C and microbial biomass, with 14 to 42% degraded over 21 days following zero-order degradation kinetics. Previous observations of AO168 degradation in environmental matrices have been highly variable, ranging from 6% over 28 days under OECD test conditions (OECD 2009) to 73% over 28 days (Gong et al. 2021). Photo-oxidative conditions will significantly increase degradation due to indirect reaction with OH radicals (SIAR 2009), although this study was conducted in dark conditions, reflecting the majority of the soil. Previous studies quantifying the AO168 degradation in soil found that rates were comparable between sterilised and unsterilised soils (Gong et al. 2021), indicating that degradation proceeds via abiotic oxidation (to AO168ox) and hydrolysis to 24DTP. The intermediate degradation product, following hydrolysis of one 24DTP group, bis(2,4-di-*tert*-butylphenyl) phosphate, was not observed, suggesting that this intermediate was rapidly further hydrolysed to 24DTP and phosphate, potentially due to the reduction in steric hindrance. The concentration of bis(2,4-di-*tert*-butylphenyl) phosphate over 28 days following the addition of 1.0 µg g^−1^ of AO168 was also low (Gong et al. 2021); therefore, it is likely it was below the limits of detection here when 0.5 µg g^−1^ of AO168 was added.

The degradation of AO168 was unaffected by measured soil properties, unlike the observed correlation of microbial biomass with DEHP and BP12 degradation, partially disproving the hypothesised order of degradation rates for BP12, DEHP and AO168 in soils with a smaller microbial community (i.e. Australian and Egyptian soils where degradation at 21 days was BP12 > AO1688 > DEHP). The different mechanisms of degradation reflect the differing chemical structure (Table 1) and therefore microbial accessibility. While phosphoesterases have been shown to degrade other organic phosphate esters (Cheng et al. 2025), the steric hindrance of 2,4-di-*tert*-butylphenyl groups may limit microbial accessibility for AO168ox. Comparably, ester and ether bonds in DEHP and BP12 are less sterically hindered and therefore more bioavailable. The differing mechanisms of degradation have significant implications for which additives may have the potential to bioaccumulate in the environment, since additives that are degraded via the microbial community may bioaccumulate in soils with a smaller microbial community, while those that degrade abiotically will not show the same pattern. Despite a lack of correlation with soil properties in this study, there were still differences in the degradation of AO168 + AO168ox between soils, with lower degradation observed in the Brazilian and Indian soils, which had contrasting properties (Fig. 2). Other environmental variables, such as temperature, moisture and UV exposure, also influence the rate of AO168 degradation in field settings (Yang et al. 2016); however, these were controlled in this study. Further work regarding the effect of soil microstructure to consider differences in additive diffusion and sorption, alongside redox conditions, is needed to determine if other soil variables which can facilitate degradation of anthropogenic organic compounds (e.g. mineral oxides (Wang et al. 2019) influence the abiotic degradation of AO168, and therefore its potential persistence in the soil environment.

### Environmental implications

The controls of soil properties on additive degradation have significant implications for the persistence of additives in soil and, ultimately, the potential for additives and associated degradation products to be taken up by plants and enter the food chain or be leached into the wider environment. This is the first study to quantify and compare the degradation of additives of different functionalities which will likely co-occur in the environment across a global transect of soils. Additives which are predominantly degraded via microbial action (DEHP and BP12) exhibited lower degradation rates in soils with lower microbial biomass and thus may have a higher potential to accumulate in soils from arid and semi-arid regions, as observed herein. Furthermore, any practices which disrupt and/or decrease microbial biomass (e.g. tillage, chronic N fertilisation) may increase the risk of additive accumulation in soil. Degradation may vary within the soil, for example, degradation may increase in the rhizosphere where microbial activity is greatest, reducing potential exposure of plant roots to additives. Conversely, additives which degrade via abiotic processes (AO168 herein) were unaffected by soil microbial biomass. Additives with different mechanisms of degradation will co-occur in plastic; therefore assessing the longevity of the chemical burden of additives from plastics in soils is complex. Ecotoxicological effects on soil microorganisms have been observed at higher concentrations for DEHP (e.g. above 20 µg g^−1^ (Wang et al. 2017) and at concentrations between 0.3 and 10 µg g^−1^ for AO168ox (Gao et al. 2024). Furthermore, degradation products of additives also pose further potential negative effects (Gao et al. 2017; Cheng et al. 2018). It is essential to consider the degradation of different additives across soil types to capture the controls on degradation and assess the potential risk of ecotoxicological effects on the soil microbial community alongside additive and degradation product accumulation in plants and organisms higher in the food chain.

## Conclusions

Here, we demonstrate the varying degradation kinetics for plastic additives shortly after leaching into agricultural soils from a global transect. This is the first study to compare the degradation of additives of different functionality in plastics, which will leach and co-occur in the environment. Key findings are:

1. DEHP, BP12 and AO168 in agricultural soils exhibited differing initial degradation kinetics, associated with different chemical functionality of the additives and soil type.
2. DEHP and BP12 degradation was linked to the size of the microbial community and available NO_3_^−^. Conversely, degradation of AO168 was not correlated with any measured soil properties, presumably due to its degradation via abiotic pathways.
3. Degradation products concentrations correlated with degraded parent additives and were not influenced by soil type.
4. The soil controls on degradation demonstrate differing potential for accumulation of plastic additives in soils with differing properties. Such soils should be targeted to determine current additive loadings and the potential ecotoxicological effects on the microbial community, as additives in these soils persist and they are most at risk of higher additive concentrations.
5. Future studies should investigate preference for microbial additive degradation in complex mixtures and the influence of UV degradation prior to entry into soil.

In summary, our results emphasise the importance of determining the degradation of organic pollutants such as plastic additives in a range of soil types, to assess the potential for bioaccumulation and subsequent ecotoxicological effects across global soils and integrate the fate of additives in soil into risk assessments for agricultural plastics.

## Supplementary Information

Below is the link to the electronic supplementary material.

ESM1 (DOCX 695 KB)

## Data Availability

Data will be made available on request.
