# Supplementary material for: Soil property controls on plasticiser, antioxidant and UV absorber additive degradation across a global soil gradient
Source: Environ Sci Pollut Res Int. 2025 Dec 4;32(57):30881–98. doi: 10.1007/s11356-025-37152-2 (PMC12811332; doi:10.1007/s11356-025-37152-2)
Supplement: Supplementary file 1 — (DOCX 695 KB) [file 11356_2025_37152_MOESM1_ESM.docx]

**Soil property controls on plasticiser, antioxidant and UV absorber additive degradation across a global soil gradient**

Michaela K. Reay^a^*, Martine Graf^b^, Maddy Murphy^a^, Charlie Monkley^a^, Perrine J. Florent^b^, Benjamin I. Collins^b^, Nguyen Van Hien^c^, Tran Minh Tien^c^, Andreia Neves Fernandes^d^, Tapan Adhikari^e^, Samantha Viljoen^f^, Mona Tolba^g,h^, Ahmed Mosa^g^, David R. Chadwick^b^, Davey L. Jones^b^, Richard P. Evershed^a^, Charlotte E.M. Lloyd^a,i^

^a^ Organic Geochemistry Unit, School of Chemistry, University of Bristol, BS8 1TS, UK;

^b^ School of Environmental and Natural Sciences, Bangor University, LL57 2UW, UK;

^c^ Soils and Fertilizer Institute, Vietnam;

^d^ Federal University of Rio Grande do Sul, Brazil;

^e^ ICAR-Indian Institute of Soil Science, India;

^f^ Bioplastics Innovation Hub, Food Futures Institute, Murdoch University, Australia;

^g^ Soils Department, Faculty of Agriculture, Mansoura University, 35516, Mansoura, Egypt;

^h^ Plant Nutrition Department, National Research Centre, Dokki, Giza, Egypt;

^i^ School of Geography, University of Bristol, BS8 1SS, UK

*Corresponding author: Michaela K. Reay, [michaela.reay@bristol.ac.uk](mailto:michaela.reay@bristol.ac.uk)

**Table S1:** Soil sampling locations and management history for soils used for additive degradation experiments.

|  | **Australia** | **Brazil** | **Egypt** | **India** | **UK** | **Vietnam** |
| --- | --- | --- | --- | --- | --- | --- |
| **Sampling location** | -31.650222, 116.696917 | 29.3956850, 50.3695929 | Composite sample from: 31.385278, 31.421667;  31.382500, 31.420833;  31.371667, 31.440833 | 23.309014 77.407731 | 53.238806, -4.015111 | 21.3481219,  106.0315539 |
| **Mean annual temperature (°C)** | 19.5 | 27.1 | 21.9 | 25.5 |  | 25.1 |
| **Cropping history** | Lupin; fallow; maize | Maize; potato | Maize, rice (summer growing season)  Sugarbeet, wheat and barley (winter season | No cultivation | Wheat, potato, grass | Spring rice-summer rice – winter corn |
| **Fertilisation summary** | 100 kg ha^−1^ Superphos | Potato and maize crop residues  Inorganic fertilizers (nitrogen-phosphorus-potassium), of 120 kg of calcitic limestone (120 kg ha^−1^) | Chicken manure/farmyard manure, Superphosphate, ammonium nitrate/urea, potassium sulphate | None | Nitrogen, phosphate, potassium applied in granule form when required | NPK compound and single N, P, K applied when needed |
| **Irrigation** | None | None | Surface irrigation | None | None | Surface irrigation |
| **Herbicide/pesticide application** | Sprayseed (paraquat and diquat)  Roundup Ultra®MAX (glyphosate) (2023) | None | None | None | Glyphosate | Sattrungdan 95WP, Boxing 99.99EW, Validacin 5SL, Hinosan 40 EC, Thiosultap-sodium, Shipper 150EC |
| **Soil sampling date** | 10/2023 | 10/2023 | 10/2023 | 11/2023 | 08/2023 | 12/2023 |

**Table S2:** Additive degradation product limits of quantification (LOQ) calculated from the calibration (range 1 ng μL^-1^ to 15 ng μL^-1^) and the *R^2^* of the calibration curve.

| **Compound** | **Source additive** | ***R^2^*** | **LOQ**  **(ng μL**^-1^**)** |
| --- | --- | --- | --- |
| **Bis(2,4-di-*tert*-butylphenyl) phosphate** | AO168 | 0.996 | 1.11 |
| **2,4-di-*tert*-butylphenol** | AO168 | 0.998 | 0.83 |
| **Benzoic acid** | BP12 | 0.956 | 3.62 |
| **Benzophenone** | BP12 | 0.967 | 1.82 |
| **2,4-dihydroxybenzophenone** | BP12 | 0.991 | 1.57 |
| **2-hydroxybenzophenone** | BP12 | 0.997 | 0.96 |
| **Dibutyl phthalate** | DEHP | 0.977 | 2.60 |
| **Diethyl phthalate** | DEHP | 0.977 | 2.60 |
| **Dimethyl phthalate** | DEHP | 0.965 | 3.25 |
| **Mono (2‑ethylhexyl) phthalate** | DEHP | 0.988 | 1.86 |
| **Phthalic acid** | DEHP | 0.971 | 4.59 |

**Table S3:** Effect of treatment and time on soil dissolved organic C (DOC), pH and electrical conductivity (EC) during the plastic additive degradation experiments determined via mixed effect models (MEMs). *p* values were calculated using the Kenward-Roger method. Significant *p* values (*p*≤0.05) are shown in **bold**. MEMs were conducted separately for each country, given differences in initial soil properties.

| **Country** | **Treatment *p* value** | | | **Timepoint *p* value** | | |
| --- | --- | --- | --- | --- | --- | --- |
|  | **DOC** | **pH** | **EC** | **DOC** | **pH** | **EC** |
| **Australia** | 0.139 | 0.276 | 0.223 | **<0.001** | 0.477 | **0.041** |
| **Brazil** | 0.511 | 0.443 | 0.606 | **<0.001** | **<0.001** | 0.225 |
| **Egypt** | 0.900 | 0.990 | 0.298 | **0.015** | **<0.001** | **0.009** |
| **India** | 0.626 | 0.405 | 0.694 | **<0.001** | **<0.001** | **0.038** |
| **UK** | 0.484 | 0.884 | 0.063 | **<0.001** | 0.504 | **0.002** |
| **Vietnam** | 0.655 | 0.476 | 0.654 | **<0.001** | **<0.001** | **<0.001** |

**Table S4:** Contributing pools to the Gram+ and fungal biomass, alongside unspecified microbial PLFAs in Table 2. Firmicute and actinobacteria PLFAs contribute to Gram+ bacteria, while Zygomycota, Ascomycota, Basidiomycota and unspecified fungal PLFAs contribute to fungal biomass. All values are mean ± SE (n=4) and b.d.l. indicates below detection limit. The *p* value is the result of One-way ANOVA, and significant values (*p*≤0.05) are indicated in bold, and different superscript letters indicate significant differences between soils, determined by post-hoc Tukey tests.

| **PLFA group** | ***p* value** | **Australia** | **Brazil** | **Egypt** | **India** | **UK** | **Vietnam** |
| --- | --- | --- | --- | --- | --- | --- | --- |
| **Firmicute PLFAs (mg kg^-1^)** | **<0.001** | 1.43 ± 0.16^e^ | 12.9 ± 0.69^a^ | 0.088 ± 0.008^e^ | 4.28 ± 0.44^d^ | 9.67 ± 0.69^b^ | 6.76 ± 0.51^c^ |
| **Actinobacteria PLFAs (mg kg^-1^)** | **<0.001** | 0.045 ± 0.005^c^ | 0.20 ± 0.02^b^ | b.d.l. | 0.16 ± 0.02^bc^ | 1.31 ± 0.07^a^ | 0.22 ± 0.02^b^ |
| **Zygomycota PLFAs (mg kg^-1^)** | **<0.001** | 0.48 ± 0.08^cd^ | 2.17 ± 0.13^b^ | 0.027 ± 0.003^d^ | 0.92 ± 0.09^c^ | 2.75 ± 0.2^a^ | 0.91 ± 0.1^c^ |
| **Ascomycota & Basidiomycota PLFAs (mg kg^-1^)** | **<0.001** | 0.062 ± 0.006^b^ | 0.58 ± 0.07^b^ | 0.062 ± 0.008^b^ | 0.37 ± 0.09^b^ | 1.31 ± 0.29^a^ | 0.29 ± 0.02^b^ |
| **Unspecified fungal PLFAs (mg kg^-1^)** | **<0.001** | 0.11 ± 0.02^c^ | 0.47 ± 0.03^a^ | 0.075 ± 0.003^c^ | 0.033 ± 0.006^c^ | 0.37 ± 0.03^a^ | 0.26 ± 0.01^b^ |
| **Unspecified PLFAs (mg kg^-1^)** | **<0.001** | 1.86 ± 0.12^b^ | 3.98 ± 0.15^a^ | 1.50 ± 0.04^b^ | 3.14 ± 0.22^a^ | 4.11 ± 0.42^a^ | 4.21 ± 0.29^a^ |

n.b. all concentrations are on a dry weight basis and corrected for the mass of added sand.

**Table S5:** Contribution of individual and total additives added to soil in relation to total soil carbon (TC) and dissolved organic carbon (DOC) at *t*=0 d.

| **Country** | **Additive (% of at *t*=0 d)** | | | | **Additive (% of DOC at *t*=0 d)** | | | |
| --- | --- | --- | --- | --- | --- | --- | --- | --- |
|  | **DEHP** | **BP12** | **AO168** | **Total** | **DEHP** | **BP12** | **AO168** | **Total** |
| **Australia** | 0.0053 | 0.0055 | 0.0056 | 0.0163 | 0.19 | 0.19 | 0.19 | 0.58 |
| **Brazil** | 0.0008 | 0.0008 | 0.0008 | 0.0024 | 0.05 | 0.05 | 0.05 | 0.16 |
| **Egypt** | 0.0184 | 0.0193 | 0.0195 | 0.0572 | 0.30 | 0.31 | 0.31 | 0.92 |
| **India** | 0.0034 | 0.0035 | 0.0035 | 0.0104 | 0.15 | 0.16 | 0.16 | 0.48 |
| **UK** | 0.0012 | 0.0012 | 0.0013 | 0.0037 | 0.09 | 0.10 | 0.10 | 0.29 |
| **Vietnam** | 0.0028 | 0.0030 | 0.0030 | 0.0088 | 0.19 | 0.20 | 0.20 | 0.60 |

**Figure S1:** Microcosm design used for the additive degradation experiment. The glass microcosms were 4 cm in diameter and 10 cm high. Soil (20 g) was added with 5% sand loaded with additives (additive treatment) or without (control) and adjusted to 30% gravimetric water content. The base was plugged with glass wool to maintain aerobic conditions throughout the soil.


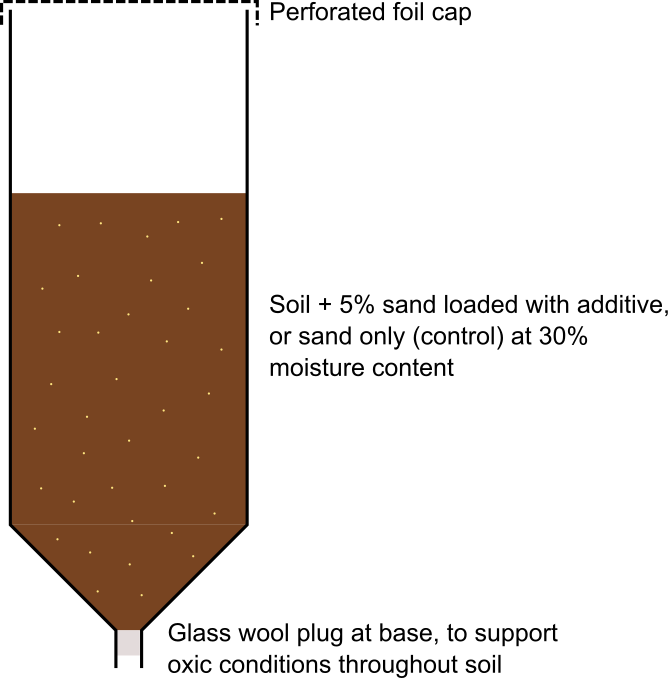


**Figure S2:** Soil pH across the 21-d degradation experiment for the additive (blue) and control (red) treatments. All values are mean ± SE (*n*=4). Where error bars are not visible, errors are smaller than the point size.


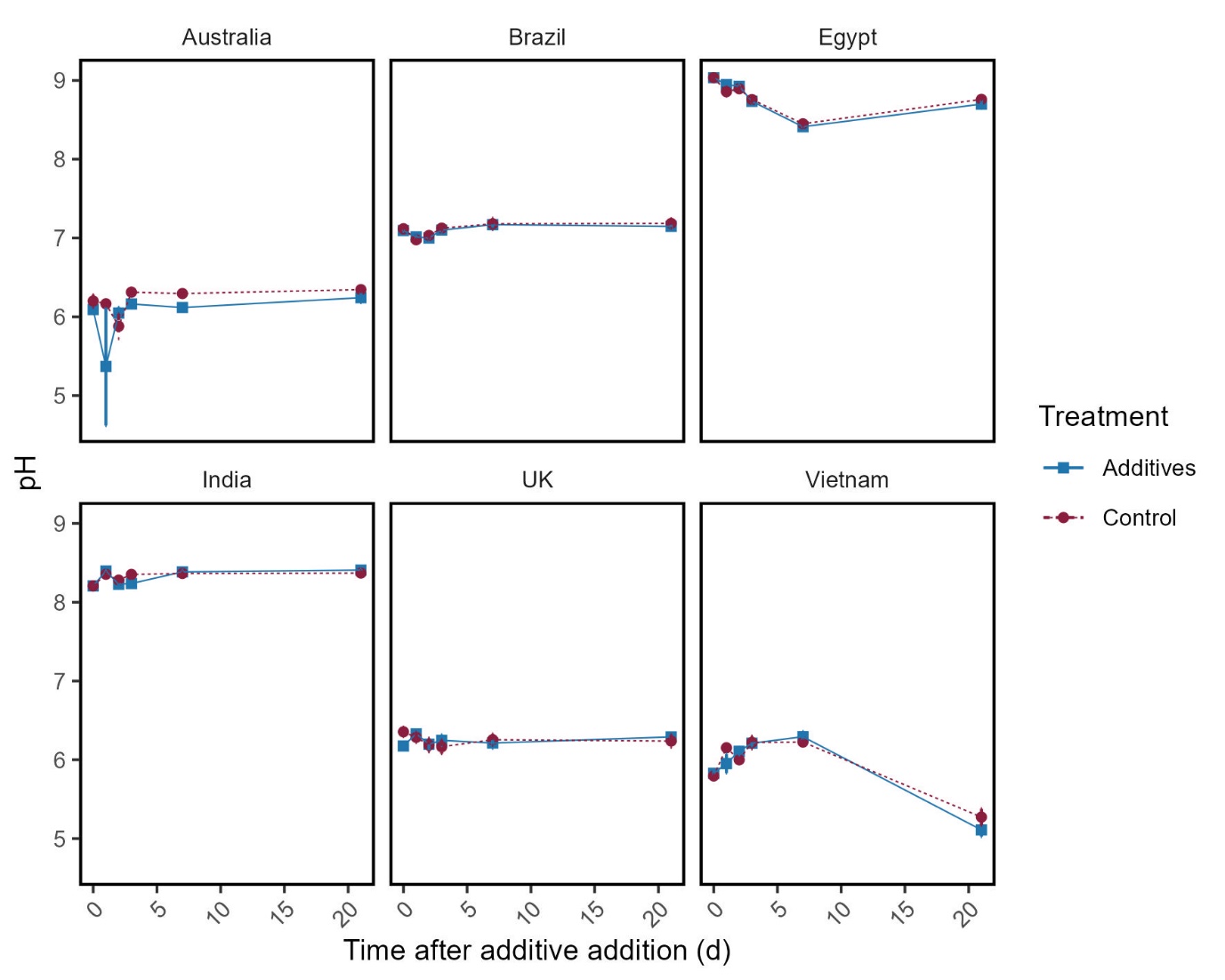


**Figure S3:** Soil electrical conductivity across the 21-d degradation experiment for the additive (blue) and control (red) treatments. All values are mean ± SE (*n*=4). Where error bars are not visible, errors are smaller than the point size.


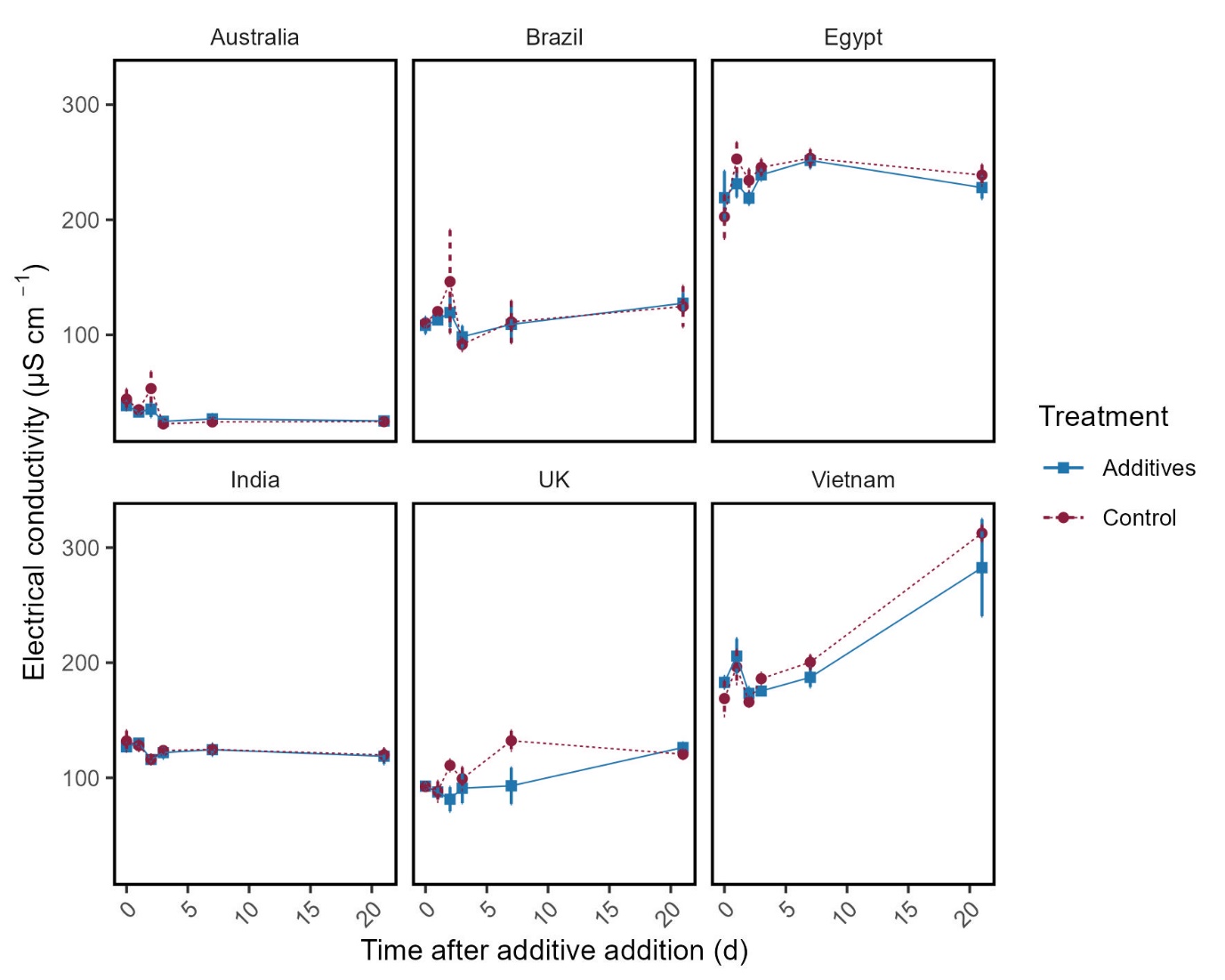


**Figure S4:** Soil DOC across the 21-d degradation experiment for the additive (blue) and control (red) treatments. All values are mean ± SE (*n*=4). Where error bars are not visible, errors are smaller than the point size.


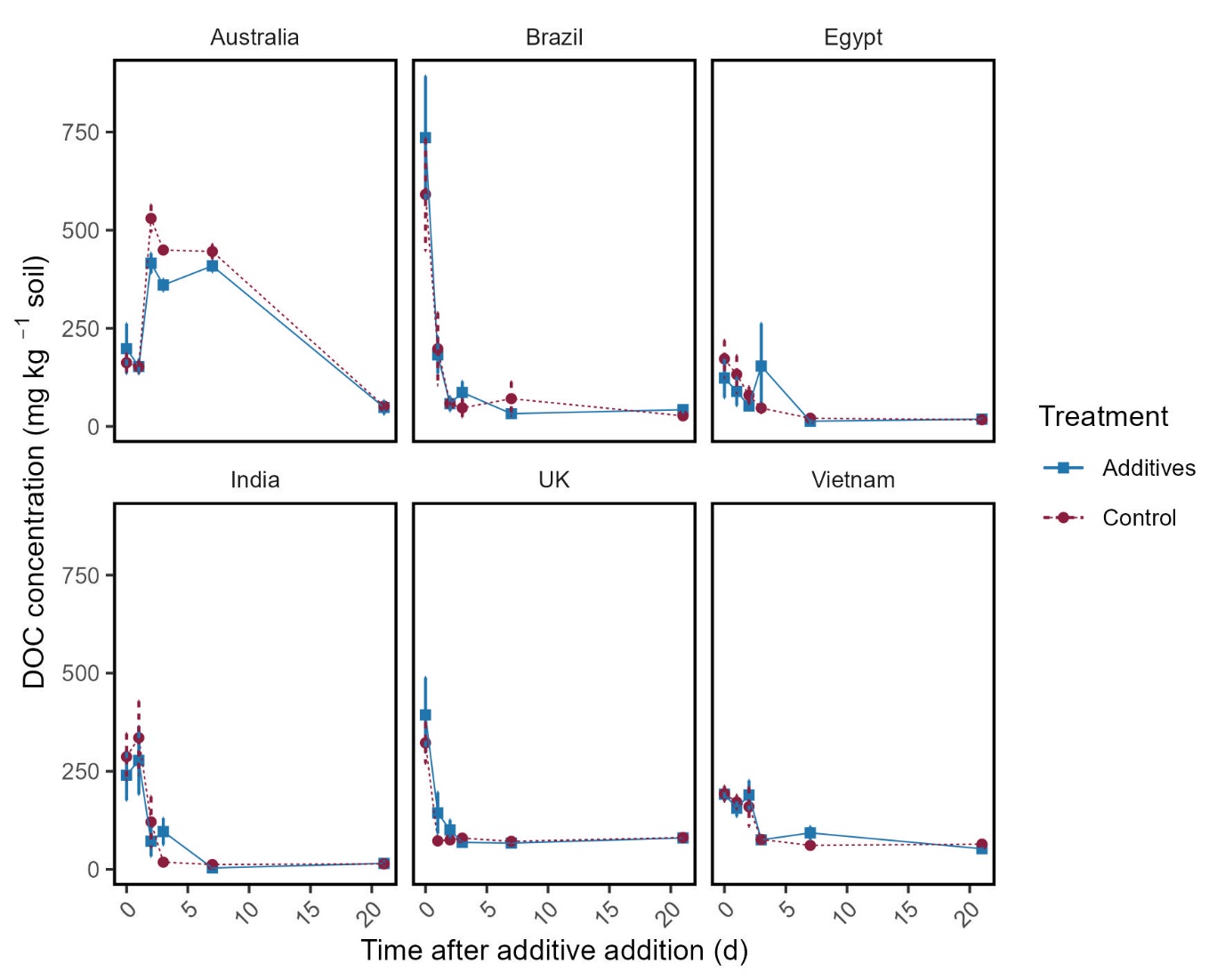


**
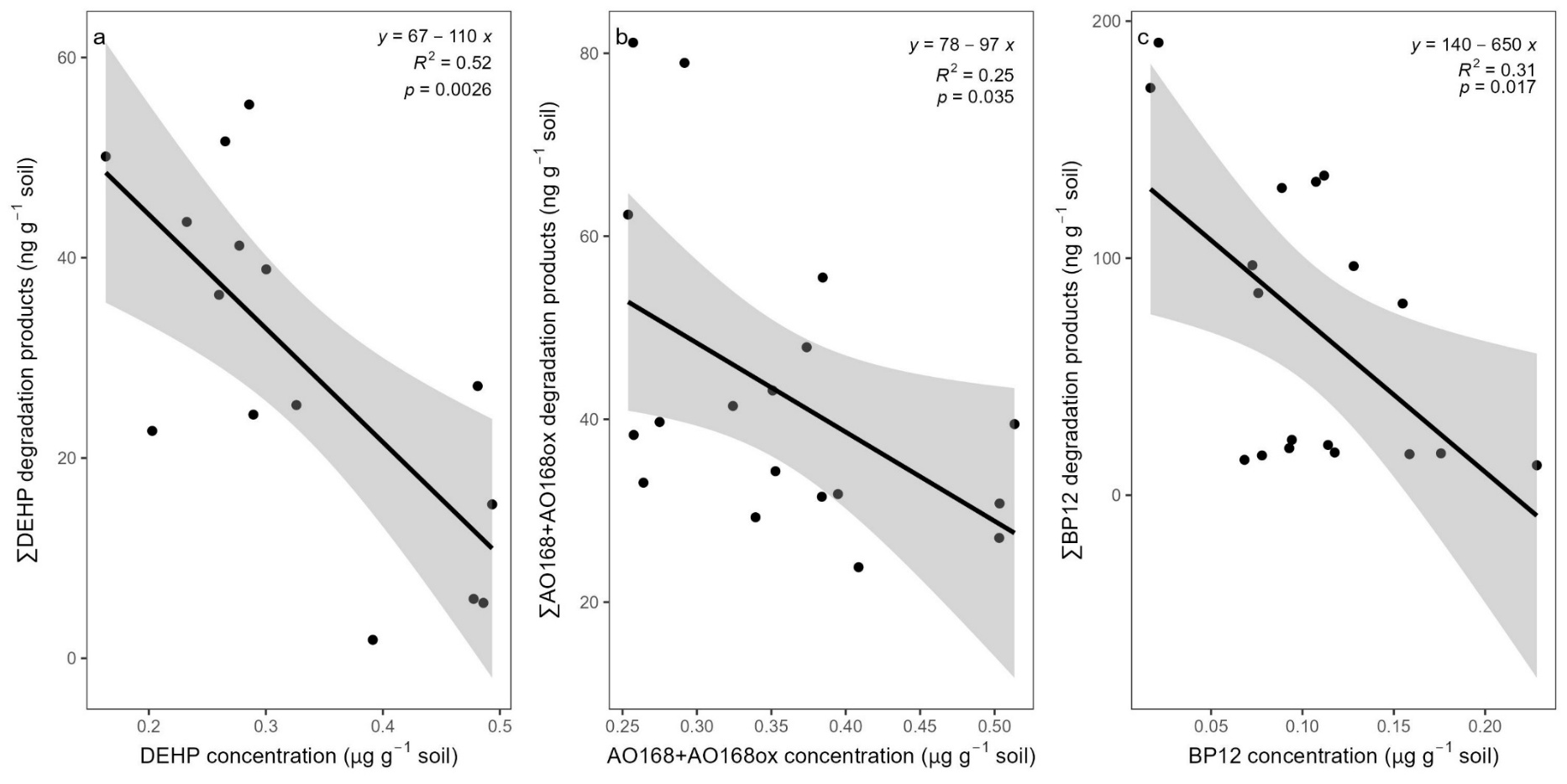
Figure S5:** Correlation of degradation products and remaining additive in soil for (a) DEHP, (b) AO168+AO168ox and (c) BP12. Lines represent linear regressions and the shading indicates 95% confidence intervals. Regression equations, R^2^ and *p*-values are inset.
